# 52500 Characterization of a Series of 1,4-diaryl-pyrazolo-pyridinones as Anti-Leishmanial Agents

**DOI:** 10.1017/cts.2021.409

**Published:** 2021-03-30

**Authors:** Hannah N. Corman, Douglas A. Shoue, Bruce J. Melancon, Mary Ann McDowell

**Affiliations:** 1 University of Notre Dame; 2 Vanderbilt University

## Abstract

ABSTRACT IMPACT: The first-line chemotherapies used to treat leishmaniasis are highly toxic intravenous antimonials yet drug resistance has begun to develop, causing the use of oral treatment options with high price tags; there is a strong need for new, safe, and effective chemotherapeutic agents to treat leishmaniasis. OBJECTIVES/GOALS: This study was conducted in order to identify novel chemical compounds that exhibit anti-leishmanial activity and to further characterize their efficacy and toxicity in in vitro and in vivo systems in the hopes of future chemotherapeutic developments. METHODS/STUDY POPULATION: A total of 28 unique 1,4-diaryl-pyrazolo-pyridinone (1,4-DAPP) compounds were synthesized and anti-leishmanial efficacy and host cell toxicity were determined using L. donovani mCherry-expressing amastigotes and THP-1 macrophages. Additional pharmacokinetic analyses of a potent 1,4-DAPP compound were conducted, revealing a potential metabolite structure. A select group of the novel compounds were screened in a cutaneous leishmaniasis (CL) murine model using L. major mCherry-expressing parasites and female Balb/C mice. The treatment consisted of 10 intralesional injections of compound over a period of 4 weeks, while lesion growth was monitored via fluorescence and manual measurements. RESULTS/ANTICIPATED RESULTS: Four experimental compounds had IC50 values less than 5 micromolar, providing similar anti-leishmanial activity to Miltefosine. Compound 9279817 had a clearance almost twice the rate of normal hepatic blood flow and had a relatively high volumes of distribution, indicating this compound is rapidly cleared and distributes into tissues. In vitro rat liver microsome assays suggest a rapid metabolism of 9279817 and MS/MS results suggest this metabolite is most likely formed via oxidation of the sulfur on the lower aryl ring. This sulfoxide metabolite has similar efficacy as the parent compound and does not exhibit toxicity in vitro. Three of the experimental compounds behaved similarly to the antimony positive control in the murine CL model. DISCUSSION/SIGNIFICANCE OF FINDINGS: This study revealed a novel structural class of compounds that have anti-leishmanial activity. Experiments show compounds with similar efficacy to Miltefosine while having significantly less cytotoxicity, suggesting that the 1,4-DAPP structural class could be further developed as a potential chemotherapeutic.

